# Adherence to diabetes quality indicators in primary care and all-cause mortality: A nationwide population-based historical cohort study

**DOI:** 10.1371/journal.pone.0302422

**Published:** 2024-05-09

**Authors:** Nura Abdel-Rahman, Orly Manor, Arnon Cohen, Einat Elran, Avivit Golan Cohen, Michal Krieger, Ora Paltiel, Liora Valinsky, Arie Ben-Yehuda, Ronit Calderon-Margalit

**Affiliations:** 1 Braun School of Public Health, Hebrew University of Jerusalem Hadassah Medical School, Jerusalem, Israel; 2 Clalit Health Services, Tel Aviv, Israel; 3 Maccabi Healthcare Services, Tel Aviv, Israel; 4 Leumit Health Care Services and Tel Aviv University, Tel Aviv, Israel; 5 Meuhedet Health Services, Tel Aviv, Israel; 6 Hadassah Medical Center, Jerusalem, Israel; National Healthcare Group, SINGAPORE

## Abstract

**Background:**

In the last three decades, much effort has been invested in measuring and improving the quality of diabetes care. We assessed the association between adherence to diabetes quality indicators and all-cause mortality in the primary care setting.

**Methods:**

A nationwide, population-based, historical cohort study of all people aged 45–80 with pharmacologically-treated diabetes in 2005 (n = 222,235). Data on annual performance of quality indicators (including indicators for metabolic risk factor management and glycemic control) and vital status were retrieved from electronic medical records of the four Israeli health maintenance organizations. Cox proportional hazards and time-dependent models were used to estimate hazard ratios (HRs) for mortality by degree of adherence to quality indicators.

**Results:**

During 2,000,052 person-years of follow-up, 35.8% of participants died. An inverse dose–response association between the degree of adherence and mortality was shown for most of the quality indicators. Participants who were not tested for proteinuria or did not visit an ophthalmologist during the first-5-years of follow-up had HRs of 2.60 (95%CI:2.49–2.69) and 2.09 (95%CI:2.01–2.16), respectively, compared with those who were fully adherent. In time-dependent analyses, not measuring LDL-cholesterol, blood pressure, HbA1c, or HbA1c>9% were similarly associated with mortality (HRs ≈1.5). The association of uncontrolled blood pressure with mortality was modified by age, with increased mortality shown for those with controlled blood pressure at older ages (≥65 years).

**Conclusions:**

Longitudinal adherence to diabetes quality indicators is associated with reduced all-cause mortality. Primary care professionals need to be supported by health care systems to perform quality indicators.

## Introduction

Diabetes has been estimated to have a global prevalence of 10.5% among adults, making it one of the most common non-communicable diseases in the world [1]. Patients with diabetes are at increased risk of developing micro- and macro-vascular complications, and they have a two- to four-fold increased risk of death compared to the general population [2], with most deaths attributed to cardiovascular diseases [3]. Diabetes care is mostly managed within the primary care setting and aims to prevent complications by controlling glucose metabolism, monitoring target organs (e.g., renal function), and treating co-existing risk factors (e.g., hypertension) [4].

In the past three decades, several programs aimed to improve primary care have implemented indicators to evaluate the quality of diabetes care [5–8]. These quality indicators mostly include process indicators, that assess the performance of various tests (e.g., testing of glycated hemoglobin-HbA1c), and intermediate-outcome indicators, that assess the achievement of certain targets (e.g., HbA1c<7%). Performance of quality indicators in diabetes has improved over the past two decades across countries [6,9–11]. Notably, the burden of diabetes care falls mainly on primary care practitioners [11] and reports on increased burden, workload and excessive managerial pressure associated with measurement of quality indicators, were published [12–14]. However, there is limited evidence whether the implementation of these programs and the associated increased performance, especially of process indicators, is associated with increased survival [15–17]. Studies that investigated the associations of intermediate-outcome indicators with mortality showed mixed results, varying from major reductions in mortality to weak or non-significant associations [2,18–25]. A previous cohort study showed that longitudinal adherence to diabetes quality indicators is associated with reduced risk of cardiac morbidity [26]. However, evidence regarding the association of longitudinal adherence to diabetes quality indicators with mortality is lacking. Therefore, we aimed to examine the association between longitudinal adherence to quality indicators and all-cause mortality among individuals with diabetes.

## Materials and methods

We conducted a nationwide historical cohort study of all adults with pharmacologically-treated diabetes in 2003–2005 in Israel (n = 222,235) and followed up to 2016. In Israel, four health maintenance organizations (HMOs) provide primary care to all permanent residents. Since 2002, these HMOs annually report to the National Program for Quality Indicators in Community Healthcare (QICH) on a predefined set of diabetes-related indicators.

To be included in the study, patients had to be 45–80 years on 1.1.2003, and to be treated with antidiabetic medications for ≥3 months in at least one of the calendar years 2003–2005. Data on quality indicators, demographic and clinical characteristics in the follow-up years were obtained from the electronic medical records of all four HMOs. The HMOs are continually updated on the vital status of their members, including the exact date of death, through linkage to the Israeli Population Registry.

### Quality indicators

Data on seven process indicators and four intermediate-outcomes were collected according to the Israeli national quality indicator set. The quality indicator set was chosen based on national and international guidelines, with a consensus of representatives from professional organizations [27]. Process indicators included annual measurements of HbA1c, LDL-cholesterol, blood pressure (BP), urinary protein, serum creatinine, ophthalmological visit, and administration of influenza vaccine. Attainment of each indicator was defined as performance at least once in a calendar year.

Intermediate-outcome indicators assessed whether patients achieved adequate control, using the last measurement in a calendar year. Two indicators were used for glycemic control. The first was an age-specific target (HbA1c≤7% for patients aged≤74 years or HbA1c≤8% for patients aged≥75 years) [28]. The second was HbA1c≤9% for all ages based on avoidance of uncontrolled diabetes. Adequate control of BP was defined as systolic BP≤140mmHg and diastolic BP≤90mmHg. For LDL-cholesterol, control was defined as ≤100 mg/dl [28].

### Covariates

The study covariates included age, ethnicity (Jewish/Arab- based on the neighborhood where the primary clinic was located), smoking (ever/never), body mass index (BMI- median weight in kg during the study period divided by height in meters squared, and categorized into <25.0, 25.0–29.9, and ≥30.0 kg/m^2^). Socioeconomic position (SEP) was defined based on the residential address, using scores (range:1–10) allocated to residential areas by the Israeli Central Bureau of Statistics [29] and updated by the POINTS Location Intelligence Company [30].

Missing data were imputed using multiple imputation by chained equations (MICE), based on strong predictors with complete data. Missing values of SEP were imputed using age and gender. Height, weight, and ethnicity were imputed using age, gender, and SEP. Smoking was imputed using age, gender, SEP, and ethnicity. The percentage of missing values were 4.5% for SEP, 8.3% for BMI, 2.5% for ethnicity, and 20.0% for smoking.

### Statistical analyses

**Fig 1:**
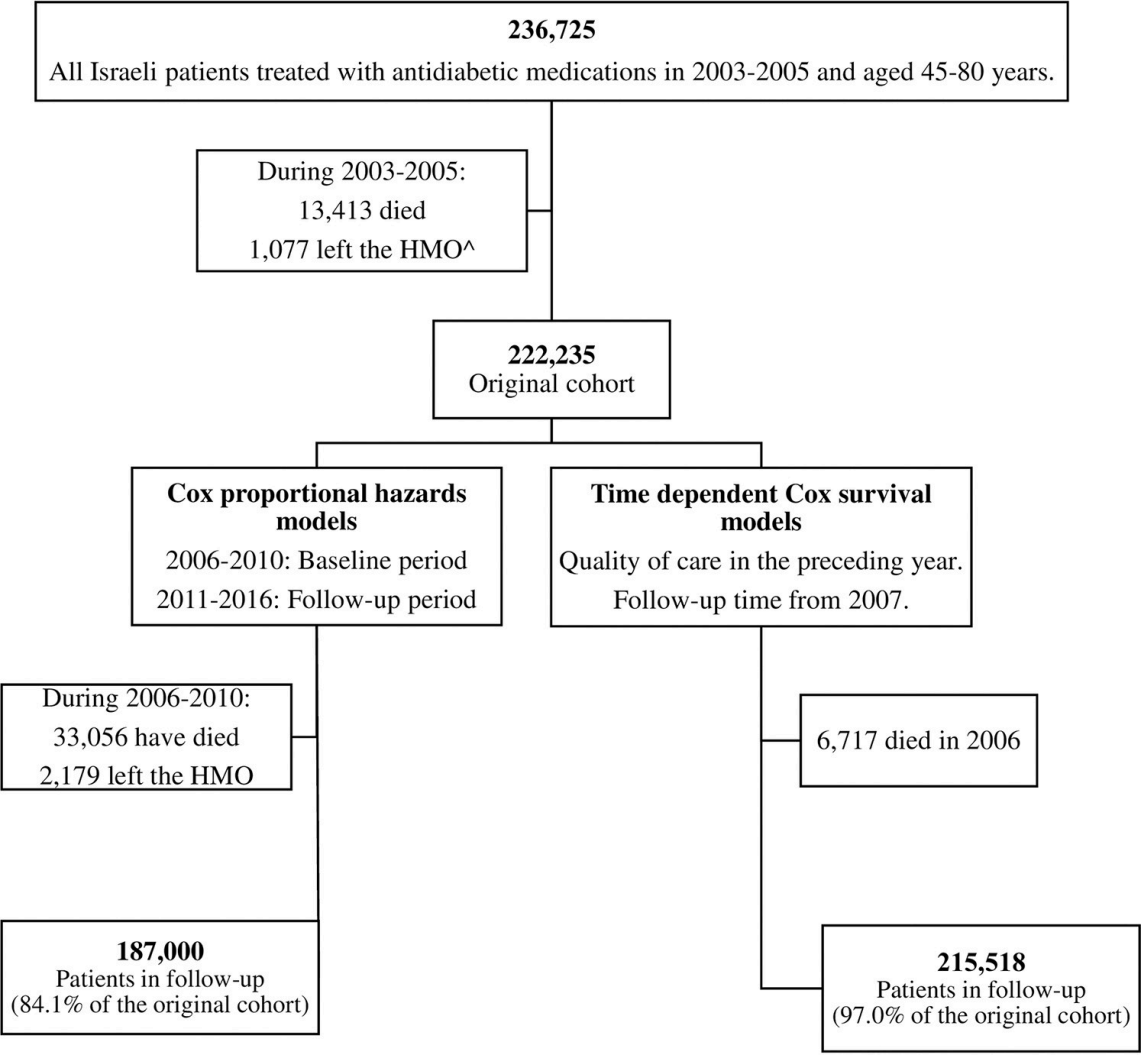
Study population and sub-population according to analyses. ^ HMO: Health maintenance organization.

The association between adherence to quality indicators and mortality was estimated using two approaches (Fig 1). First, the study period was divided into a baseline period) 2006-2010 for adherence assessment), and a follow-up period) 2011-2016 for outcome assessment). For each calendar year, indicators were dichotomized, scoring 1 if the indicator was attained and 0 otherwise. For intermediate-outcome indicators, non-performance was coded as non-attainment and received a value of 0. The degree of adherence to each quality indicator was defined as the number of years in which the indicator was attained in the baseline period (scoring 0–5). In addition, a composite score was calculated for each year, summing the total number of performed process indicators per year, ranging from 0 (none) to 7 (all). Then, an average composite score over the baseline five-years was calculated. These analyses included patients who survived until 2010 (n = 187,000). S1 Table presents the baseline characteristics of patients who were included in these analyses compared to those who died in 2006–2010 (n = 35,235). Follow-up time was calculated from 1.1.2011, to date of death, changing HMO (2.0%) or end of follow-up (31.12.2016), whichever occurred first. Cox proportional hazards models were used to estimate hazard ratios (HRs) and 95%CIs for the associations between adherence to quality indicators and mortality. All models were adjusted for age, gender, smoking, BMI, SEP and HMO. We confirmed the proportional hazards assumption by inspection of log-minus-log plots. For one of the HMOs (8% of the study population), documentation of BP and influenza vaccination was missing during the baseline period, thus members of this HMO were excluded from the sensitivity analysis.

The second approach took into account the year-by-year changes in the attainment of quality indicators during the entire decade (2006–2016). In this analysis, the combined effect of measuring and achieving adequate control was estimated for each of the three indicators: HbA1c, BP and LDL-cholesterol. Each combined indicator was categorized into unmeasured, uncontrolled, or controlled. For these analyses, follow-up time was counted from 1.1.2007, attributing deaths to quality of care in the preceding year, to avoid reverse causality. The analyses included patients who lived through 2006 (n = 215,518). Time-dependent Cox models were constructed with the annual combined indicators as time-dependent variables. End of follow-up time and the covariates adjusted for in modelling were similar to those used in the first approach.

All statistical analyses were carried out using RStudio (version 3.5.1(. P value<0.05 were considered to be statistically significant.

Ethical approvals were obtained from the institutional review boards of all four HMOs: Clalit Health Services (0132-17-com2), Maccabi Health Services (0119-17-BBL), Meuhedet Health Services (03-02-10-17) and Leumit Health Services (0237-17-LEU). All data for this retrospective study were fully anonymized and the four institutional review boards waived the requirement for informed consent on the basis of preserving participants’ anonymity.

## Results

Table 1 presents the characteristics of the study population. At baseline (January 2006), participants were 65.8 years old (SD:9.3), 51.7% were women, and 35.5% had been previously diagnosed with heart disease.

**Table 1:** Baseline characteristics of the study population, according to vital status by the end of follow-up (2006–2016).

Variable	Total (n = 222,235)	Alive (n = 142,686)	Died (n = 79,549)
**Female sex**	51.7	52.2	50.7
**Age**, years (mean±SD)	65.8 ±9.3	62.8 ±8.4	71.1 ±8.3
**Arabs**	10.5	14.0	4.1
**Socioeconomic position**			
1–2 (low)	4.1	4.0	4.3
3–5	48.5	46.9	51.4
6–8	41.2	42.4	38.9
9–10 (high)	6.2	6.7	5.4
**Smokers**	29.7	31.0	27.4
**BMI**, kg/m^2^ (mean±SD)	30.2±6.2	30.5±5.9	29.7±6.7
**Comorbidities**			
Heart disease	35.5	27.1	50.1
Advanced eye disease	20.4	16.1	27.9
End stage renal disease	0.88	0.22	2.05
Lower limb amputation	1.0	0.39	2.2
**Performance of process indicators in 2006**			
HbA1c testing	88.1	89.8	85.1
LDL-cholesterol testing	87.7	89.1	85.4
Blood pressure measurement	77.5	77.5	77.5
Serum creatinine testing	90.3	90.0	90.9
Urinary protein testing	67.2	71.3	60.1
Eye clinic visit	59.6	62.1	55.1
Influenza vaccination	35.1	32.7	39.3
All process indicators	17.0	17.2	16.7
**Performance of intermediate-outcome indicators: achieving target goals in 2006**				
HbA1c ≤9%	76.2	78.4	72.3
HbA1c ≤7% or ≤8%*	46.5	45.3	48.5
LDL-cholesterol ≤100 mg/Dl	48.8	49.6	47.4
Blood pressure ≤140/90 mmHg	54.8	56.1	52.6

Values are expressed as percent except where otherwise is stated. BMI: Body mass index. Heart disease included ischemic heart disease and heart failure. Advanced eye disease included retinopathy, impaired or loss of vision. HbA1c: Glycated hemoglobin, LDL-cholesterol: Low density lipoprotein cholesterol. HbA1c ≤9%. * HbA1c ≤7% for patients aged ≤74 years and HbA1c ≤8% for patients aged ≥75 years.

During 2,000,052 person-years of follow-up (median:11 years), 79,549 (35.8%) patients died, yielding an incidence rate of 39.8 per 1,000 person-years. People who died were older and had higher prevalence of comorbidities than those who survived (Table 1).

### Process indicators

More than 70% of the participants had annual testing of HbA1c, LDL-cholesterol or creatinine, in all-five years between 2006 and 2010. A slightly lower proportion had recorded measurements of BP (64.7%) and substantially lower proportion (<40%) for assessment of proteinuria, ophthalmological visit, or influenza vaccinations (Table 2; see S2 Table for baseline characteristics by adherence). During the study period, the annual performance rates increased, with the most noticeable improvements in influenza vaccination and BP measurements (S1 Fig). Inverse dose-response associations between degree of adherence and mortality was shown for most process indicators (except for creatinine and influenza vaccination), with significant inverse linear trends (Table 2). The strongest inverse associations were noted for testing of urinary protein and for ophthalmological visits. Participants who were not tested for proteinuria in any of the baseline years had significantly higher risk for mortality, with HR of 2.60 (95%CI:2.49–2.69) compared with those who were adherent in all-5-years (Table 2). Those who did not visit an ophthalmologist in any of these years had a HR for mortality of 2.09 (95%CI:2.01–2.16) compared with those who were adherent in all-5-years (Table 2). Incorporating all process indicators into a composite score demonstrated that performance of any additional indicator was associated with a 16% reduced risk for mortality (HR:0.84, 95%CI:0.84–0.85).

**Table 2:** All-cause mortality (2011–2016) by degree of adherence to process indicators (2006–2010), N = 187,000.

		Degree of adherence- No. of years the quality indicators was performed					
		0	1	2	3	4	5
**Quality indicator**							
HbA1c testing*	%	1.2	1.2	2.4	5.9	16.4	72.8
	**HR** (95% CI)	**1.13** (1.02–1.25)	**1.53** (1.41–1.65)	**1.48** (1.39–1.56)	**1.41** (1.36–1.46)	**1.26** (1.23–1.29)	**1**
LDL-cholesterol testing*	%	0.9	1.2	2.6	6.3	18.0	71.0
	**HR** (95% CI)	**1.31** (1.16–1.46)	**1.72** (1.59–1.87)	**1.61** (1.53–1.71)	**1.50** (1.43–1.54)	**1.24** (1.21–1.27)	**1**
Blood Pressure measurement*	%	3.1	5.8	2.3	6.2	17.9	64.7
	**HR** (95% CI)	**1.58** **(**1.49–1.67)	**1.42** (1.36–1.49)	**1.59** (1.50–1.68)	**1.20** (1.16–1.25)	**1.06** (1.04–1.09)	**1**
Serum creatinine testing	%	0.7	0.9	2.1	5.5	16.8	74.0
	**HR** (95% CI)	**0.95** (0.82–1.09)	**1.21** (1.09–1.35)	**1.21** (1.12–1.29)	**1.06** (1.02–1.11)	**1.00** (0.97–1.02)	**1**
Urinary protein testing*	%	3.7	4.8	9.0	16.7	28.4	37.4
	**HR** (95% CI)	**2.60** (2.49–2.69)	**2.18** (2.10–2.26)	**1.77** (1.72–1.83)	**1.42** (1.39–1.46)	**1.16** (1.13–1.19)	**1**
Eye clinic visit*	%	6.5	10.3	15.3	19.9	21.9	26.1
	**HR** (95% CI)	**2.09** (2.01–2.16)	**1.73** (1.68–1.79)	**1.48** (1.44–1.52)	**1.30** (1.27–1.34)	**1.14** (1.11–1.18)	**1**
Influenza vaccination	%	30.9	11.0	9.8	10.0	13.6	24.7
	**HR** (95% CI)	**1.06** (1.03–1.09)	**1.16** (1.12–1.20)	**1.19** (1.15–1.23)	**1.22** (1.18–1.26)	**1.18** (1.15–1.21)	**1**

HbA1c: Glycated hemoglobin, LDL-cholesterol: Low density lipoprotein cholesterol, HR: Hazard ratio, CI: Confidence interval. *Significant inverse linear trend in HRs. Models were adjusted for age, gender, body mass index, socioeconomic position, smoking and health maintenance organization. In all analyses, 5 years of adherence were the reference group, hence the HR equals 1.

Excluding patients for whom documentation of BP and influenza vaccination were unavailable for the baseline period did not materially change the results (S3 Table).

### Intermediate-outcome indicators

**Fig 2:**
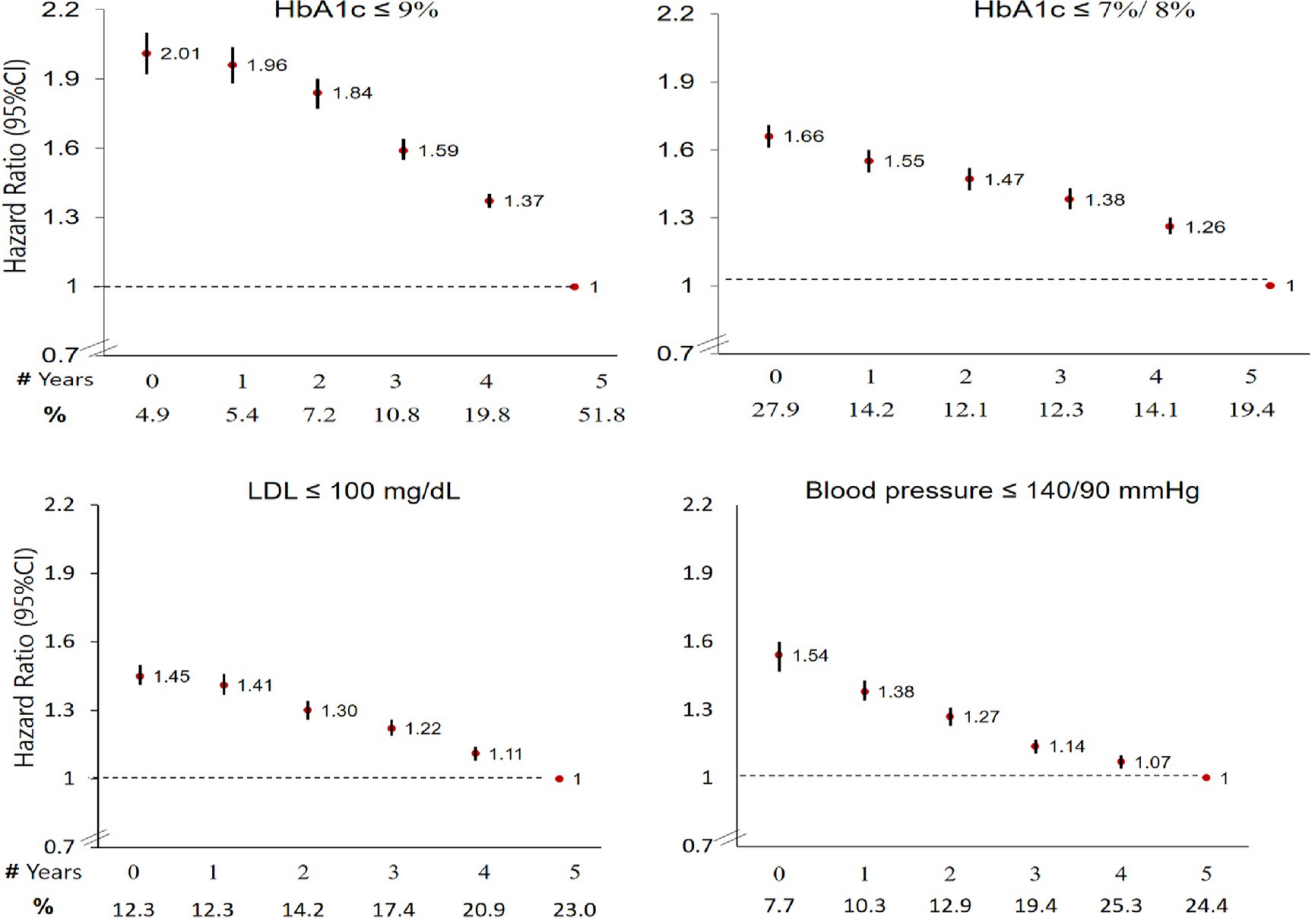
All-cause mortality (2011–2016) by degree of adherence to intermediate-outcome indicators (2006–2010), N = 187,000. Degree of adherence to intermediate-outcome indicators: Number of years with achieved target level, in 2006–2010. Lack of measurement was considered as uncontrolled, 0 (never controlled during 2006–2010) and 5 (controlled in each year). HbA1c: Glycated hemoglobin ≤7% among patients aged ≤74 years or HbA1c ≤8% among patients aged ≥75 years, HbA1c <9%, LDL: Low density lipoprotein cholesterol. Circles denote hazard ratio, and horizontal lines represent 95% CIs. Models were adjusted for age, gender, body mass index, socioeconomic position, smoking and health maintenance organization.

Among the study population, 19.4–24.4% achieved the target levels of HbA1c (≤7/8%), LDL-cholesterol (≤100 mg/dL), or BP (≤140/90 mmHg) every year in 2006–2010 (Fig 2; see S4 Table for baseline characteristics of the study population by indicator-attainment in 2006). Patients who failed to achieve these target levels in all-5-years had similarly increased risks of mortality (HbA1c:HR 1.66 (95%CI:1.61–1.71); LDL-cholesterol:1.45 (1.41–1.50); BP:1.54 (1.47–1.60)). Patients with uncontrolled diabetes (HbA1c> 9%) in all-5-years had twice the risk of mortality compared with those who had HbA1c≤9% in all years (HR:2.01, 95%CI:1.92–2.10), with a monotonic decline in risk for each additional year within the target level (Fig 2). Including the three indicators in one model slightly attenuated these associations (S5 Table). Associations between attainment of intermediate-outcome indicators and mortality were not modified by gender, age, SEP or heart disease (S6–S9 Tables).

To estimate the role of a potential survival bias in the associations between adherence to quality indicators and mortality, we conducted analyses using the 2006 adherence as exposure among patients who survived 2006 (n = 215,518) and among those who survived 2010 (n = 187,000). These models yielded similar results, suggesting that survival bias did not account for our results (S10 Table).

### Combined indicators: Time dependent analyses

**Fig 3:**
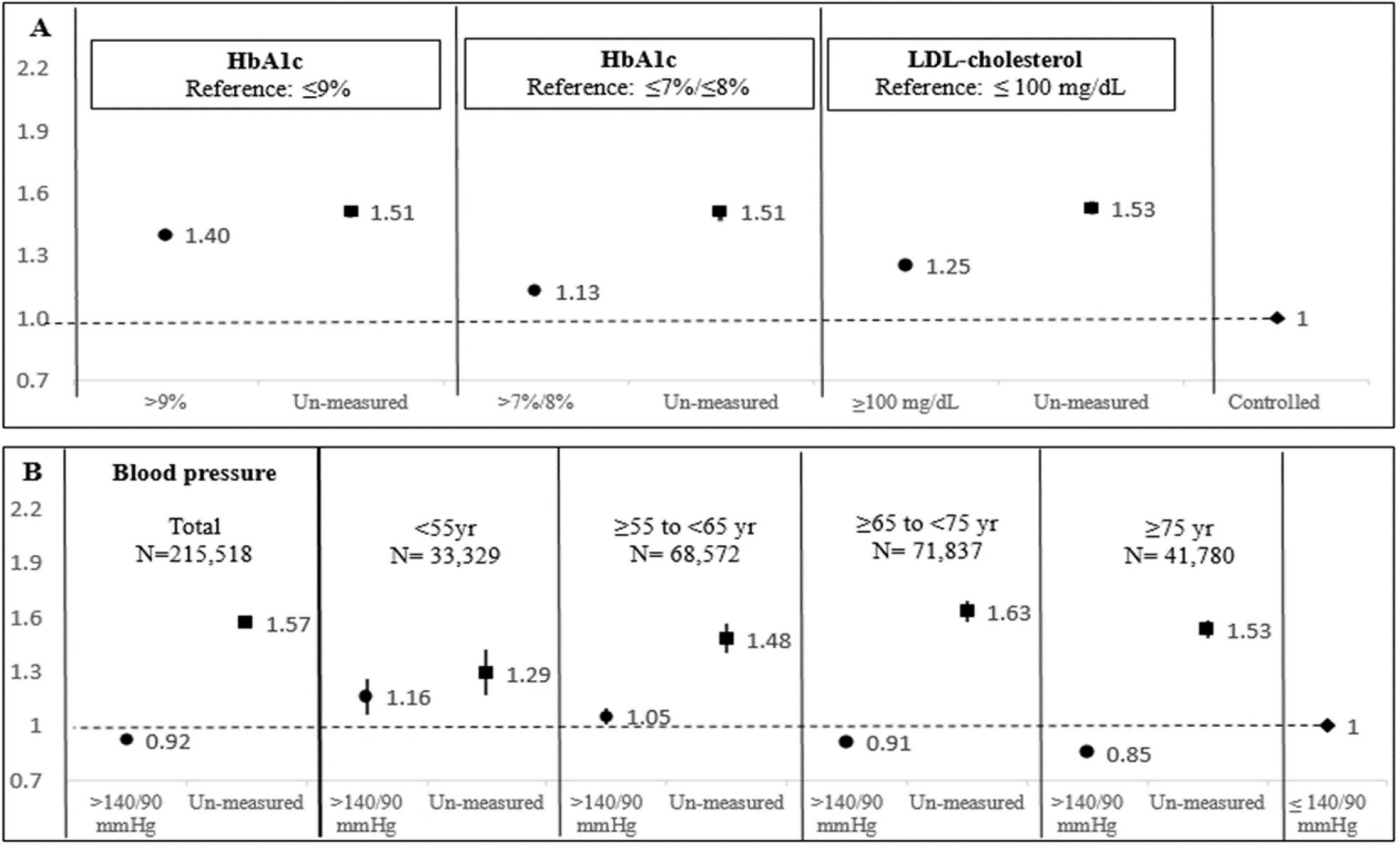
Adjusted hazard ratio (95% CI) for mortality using time dependent models, N = 215,518. A-Three models for HbA1c and LDL- cholesterol. B- Five models for blood pressure, total population and stratified models by age group in 2006. Reference group in all the models is adequate control, circles denote un-controlled and squares denote un-measured. Models were adjusted for age, gender, body mass index, socioeconomic position, smoking and health maintenance organization. HbA1c: Glycated hemoglobin, HbA1c≤9% for all ages, HbA1c ≤7% among patients aged ≤74 years or HbA1c ≤8% among patients aged ≥75 years, LDL-cholesterol: Low density lipoprotein cholesterol.

To take into account the year-by-year changes in adherence to both process and intermediate-outcome indicators throughout the whole study period, we conducted a time-dependent analysis, investigating separately HbA1c, LDL-cholesterol, and BP Compared with achieving control of HbA1c (HbA1c≤7/8%), not being tested for HbA1c was associated with HR of 1.51 (95%CI:1.47–1.54), and inadequate control was associated with HR of 1.13 (95%CI:1.11–1.15) (Fig 3A). Similar results were found for LDL-cholesterol (Fig 3A). In these analyses, HbA1c>9% was associated with HR of 1.40 (95% CI: 1.37–1.43).

Not-measuring BP was associated with a similarly increased risk (HR:1.29–1.63) in all age groups. However, having BP>140/90mmHg showed differential association with mortality by age, with increased risk among individuals<65 years and decreased risk among individuals≥ 65 year; HRs decreased with age from 1.16 to 0.85 (Fig 3B).

Sensitivity analyses that included comorbidities as time dependent variables did not materially change these results (S2 Fig).

## Discussion

In this large population-based historical cohort study, adherence to diabetes quality indicators was associated with reduced risk of all-cause mortality. A key finding of this study was the inverse, dose-response association between adherence to quality indicators and mortality, which held true for both process indicators (except for creatinine and influenza vaccination) and intermediate-outcome indicators. Our results suggest that control of HbA1c and LDL-cholesterol are associated with increased survival, whereas the association of controlled BP is modified by age.

Our results on process indicators suggest that performance of each additional indicator is associated with a 16% reduction in the risk of mortality. These results are in agreement with a previous study that showed an incremental reduction of 14% with the addition of each quality indicator [31]. Nevertheless, our results suggest that the magnitude of reduction is not uniform across all process indicators. The strongest associations were detected for measurement of proteinuria and ophthalmological visit. It should be noted that these two indicators were among those with the lowest rates of adherence at baseline, suggesting that these indicators better reflect the quality of diabetes-oriented care, rather than the general utilization of healthcare services. While reduction of albuminuria that follows assessment of proteinuria could indeed lower the risk of mortality [32], adherence to ophthalmological exams has been associated with increased adherence to treatment and improved diabetes education [33]. Thus, the strong association may reflect a more global assessment of the patient’s and physician’s adherence to diabetes care. And this may indeed be true for our findings that not being tested for HbA1c or LDL-cholesterol or BP was associated with higher mortality risk than having inadequate control.

This study found that not being tested for HbA1c or LDL-cholesterol were associated with 50% increased hazard for mortality. There is limited previous evidence on the association of performance of specific tests and mortality. A previous study in the setting of Medicare has suggested that measurement of HbA1c was associated with macro-vascular complications, but not with mortality, probably due to short follow up [15]. A study supporting our finding on LDL-cholesterol testing showed that adults with diabetes who did not perform lipid tests in a 24-months were at least 1.5 times more likely to die from cardiovascular disease compared to patients who were tested [16].

In this study, poor glycemic control was strongly and significantly associated with mortality.

Patients who failed to achieve HbA1c≤ 9% or HbA1c≤ 7/8% in any of the baseline years had 2- or 1.7-folds increased hazard for mortality, respectively. This finding is in accordance with previous studies [18,19,34–39], that showed that inadequate control of HbA1c was associated with increased risk of mortality [e.g., HbA1c>9% associated with HR of 1.78 [36]]. However, these previous studies did not address the degree of adherence over the years. Previous studies support our finding on the association of control of LDL-cholesterol and reduced mortality [20,34,36,40,41]. A meta-analysis showed a 9% proportional reduction in mortality per mmol/L reduction in LDL-cholesterol [41]. However, contradicting results for both inadequate control of HbA1c and LDL-cholesterol were shown in a large cohort study (n = 859,617), where both HbA1c and LDL-cholesterol were not associated with mortality [21].

Our findings suggest that the association between BP target achievement and mortality is age-dependent. Inadequate control of BP (>140/90mmHg) was associated with higher mortality risk among participants aged<65, but with surprisingly significant lower mortality risk among participants aged≥65 years. A meta-analysis of participants in clinical trials supported our finding regarding patients aged<65, and showed that lower systolic BP was associated with lower risk of mortality [42]. Regarding older people with diabetes, previous studies have shown that seemingly controlled systolic BP was associated with increased mortality [43–46]. The reason for this increased risk remains unclear; it has been hypothesized, that low BP in older patients could lead to ischemic events or indicates a worse health status (e.g., comorbidities or malnutrition) [47,48]. Indeed, the International Diabetes Federation recommended a more lenient BP target (<150/90mmHg) for patients aged >80 years [49]. Future studies are needed to support our finding and establish the optimal BP target level for patients aged 65 years and older.

Our study has some limitations. First, the study did not include persons with diabetes who were managed by lifestyle modifications alone, calling into question the generalizability of our study results. Notably, documentation of laboratory results (HbA1c and glucose) and physician diagnoses during 2003–05 was found as a poor-quality data, while the quality of data on purchase of antidiabetic medications was high. Furthermore, the latter definition, has a high specificity of diabetes and includes the majority (85% based on Quality Indicators in Community Healthcare data) of persons with diabetes. Second, this study cannot distinguish between diabetes care per-se and individuals’ characteristics and behaviors, it could be that healthier patients are more adherent to quality indicators, or that patients who are adherent to quality indicators tend to be more adherent to other health advice (e.g., physical activity and diet) that we did not measure. However, our results showed that BMI and smoking rates were similar among adherent and non-adherent patients, furthermore models were adjusted for several important health-related variables (smoking, BMI, age, gender and SEP), and presence of comorbidities was taken into account in the sensitivity analyses. Moreover, our results on process indicators suggest that the associations were not uniform across all process indicators. Third, data regarding type of diabetes and duration of disease were missing. Our age restriction minimized the proportion of patients with type 1 diabetes in the cohort. Fourth, the Israeli national quality indicator set does not cover all aspects of diabetes care (e.g., foot care) and differs somewhat from international guidelines [28].

Our study’s strengths include its comprehensiveness in terms of a national coverage of all Israeli patients with pharmacologically-treated diabetes, reducing the probability of selection bias, and the assessment of numerous quality indicators enabling a comparison of the benefit of adherence to each of these indicators. Second, the study has the advantage of evaluating the associations between adherence to quality indicators, and patient’s health outcomes within a real-life setup, i.e. actual care that patients received in the real-world setting and not a care assigned by trial protocol. Clinical trials are of high importance in providing evidence-based data for the development of quality indicators, yet they may suffer from limitations regarding generalizability. Third, to the best of our knowledge, this is the first study that estimated the associations between degree of adherence over a number of years and mortality. Fourth, associations were estimated using two statistical approaches and findings were robust. Finally, the large study population allowed us to explore whether the associations were modified by gender, age, presence of cardiac disease or SEP.

In conclusion, our study shows that longitudinal adherence to diabetes quality indicators in the primary care setting, is associated with reduced mortality among people with diabetes; it is therefore worth the effort invested by primary care practitioners in the performance of quality indicators. Furthermore, primary care professionals need to be supported by healthcare systems while performing quality indicators, given their demonstrated association with increased survival. Quality-of-care programs that increase the performance of quality indicators are probably effective in improving health outcome among people with diabetes.

## Supporting information

S1 Checklist STROBE statement—checklist of items that should be included in reports of *cohort studies*. (DOCX)

S1 Fig Attainment of quality indicators in 2006 and 2015^a^. (TIF)

S2 Fig Adjusted hazard ratio (95% CI) for mortality (2007–2016) by the combined indicator of blood pressure using time dependent models, taking into account the presence of comorbidities as a time dependent variable, N = 215,518. (TIF)

S1 Table Baseline characteristics according to follow-up period. (DOCX)

S2 Table Baseline characteristics of the study population by adherence to process indicators in 2006*. (DOCX)

S3 Table Adjusted hazard ratio (95% CI) for mortality while excluding patients from the health maintenance organization in which documentations of blood pressure and influenza vaccination were unavailable for the baseline period, (N = 174,327). (DOCX)

S4 Table Baseline characteristics of the study population by adherence to intermediate-outcome indicators in 2006*. (DOCX)

S5 Table Adjusted hazards ratio (95% CI) for mortality (2011–2016) by number of years with achieved target level (2006–2010). All the intermediate-outcome indicators in the same model, (N = 187,000).
(DOCX)

S6 Table Adjusted hazards ratio (95% CI) for mortality by number of years with achieved target level (2006–2010), stratified by gender N_Total_ = 187,000, N_Female_ = 97,134, N_Male_ = 89,866. (DOCX)

S7 Table Adjusted hazards ratio (95% CI) for mortality by number of years with achieved target level (2006–2010), stratified by age group, N_Total_ = 187,000, N_age<65_ = 95,619, N _age≥65_ = 91,381. (DOCX)

S8 Table Adjusted hazards ratio (95% CI) for mortality by number of years with achieved target level (2006–2010), stratified by presence of cardiac disease. (DOCX)

S9 Table Adjusted hazards ratio (95% CI) for mortality by number of years with achieved target level (2006–2010), stratified by socioeconomic position. (DOCX)

S10 Table Adjusted hazards ratio (95% CI) for mortality (2007–2016) by the combined indicators in 2006, (A) among patients who were in follow-up and survived 2006 and (B) among those who were in follow-up and survived 2010. (DOCX)
