# Supplementary figures and images for: Adherence to diabetes quality indicators in primary care and all-cause mortality: A nationwide population-based historical cohort study
Source: PLoS One. 2024 May 9;19(5):e0302422. doi: 10.1371/journal.pone.0302422 (PMC11081362; doi:10.1371/journal.pone.0302422)

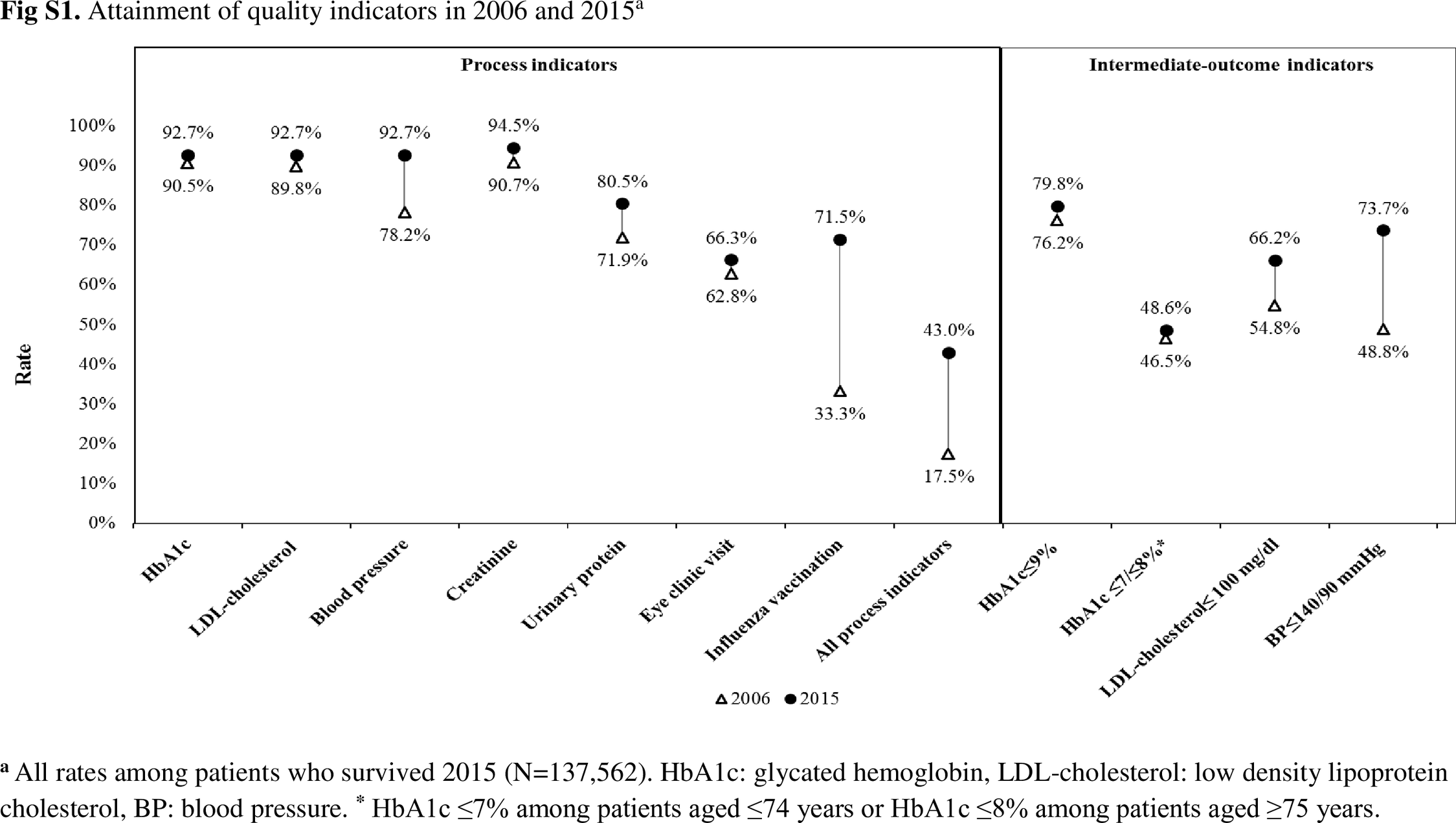

Supplement: S1 Fig — (TIF) [file pone.0302422.s002.tif]

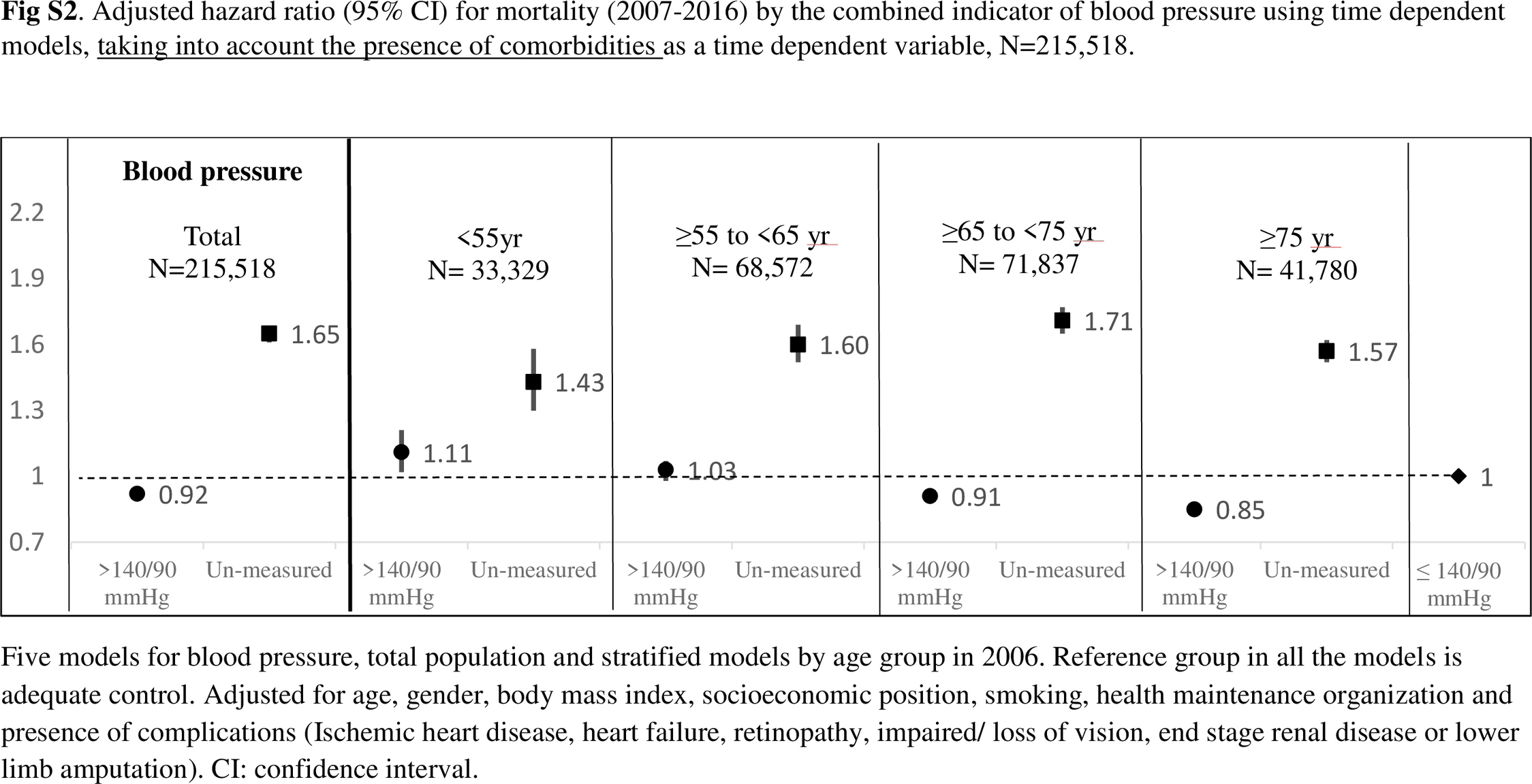

Supplement: S2 Fig — (TIF) [file pone.0302422.s003.tif]
