# Supplementary material for: Adherence to diabetes quality indicators in primary care and all-cause mortality: A nationwide population-based historical cohort study
Source: PLoS One. 2024 May 9;19(5):e0302422. doi: 10.1371/journal.pone.0302422 (PMC11081362; doi:10.1371/journal.pone.0302422)
Supplement: S1 Table — (DOCX) [file pone.0302422.s004.docx]

**Table S1.** Baseline characteristics according to follow-up period*

| **Variable** |  | Patients who died or left the HMO  between 2006-2010 | Patients who survived 2010 |
| --- | --- | --- | --- |
| N |  | 35,235 **^a^** | 187,000 |
| Female sex (%) |  | 50.3 | 51.9 |
| Age (years) |  | 71.6±8.4 | 64.7±9.0 |
| Socioeconomic position |  |  |  |
| 1-2 (low) |  | 4.4 | 4.0 |
| 3-5 |  | 53.1 | 47.6 |
| 6-8 |  | 37.5 | 41.9 |
| 9-10 (high) |  | 4.9 | 6.5 |
| Ever smokers (%) |  | 26.2 | 30.4 |
| BMI (kg/m^2^) |  | 29.4±6.1 | 30.4±6.2 |
| Overweight (%) |  | 40.6 | 38.2 |
| Obese (%) |  | 26.7 | 29.1 |
| **Process indicators; % performed in 2006** | | | |
| HbA1c |  | 78.6 | 90.0 |
| LDL-cholesterol |  | 79.8 | 89.3 |
| Blood pressure |  | 71.8 | 78.6 |
| Serum creatinine |  | 88.2 | 90.7 |
| Urinary protein |  | 50.4 | 70.5 |
| Eye clinic visit |  | 46.2 | 62.2 |
| Influenza vaccination |  | 35.0 | 35.1 |
| All process indicators |  | 12.3 | 17.9 |
| **Intermediate-outcome indicators; %controlled in 2006** | | | |
| HbA1c (≤9%) |  | 66.3 | 78.1 |
| HbA1c (≤7%/ ≤8%) ^#^ |  | 33.4 | 43.3 |
| LDL- cholesterol ≤100 mg/dL |  | 36.1 | 38.6 |
| Blood pressure ≤140/90 mmHg |  | 50.3 | 55.7 |

* Values are expressed as percent except for plus–minus values are means ±SD. ^a^ 33,056 died during 2006-2010 and 2,179 left the health maintenance organization. HMO: health maintenance organization, BMI: body mass index, Overweight: BMI 25.0-29.9, Obese: BMI ≥30.0 kg/m^2^, HbA1c: glycated hemoglobin, LDL-cholesterol: low density lipoprotein cholesterol. ^#^ HbA1c ≤7% for patients aged ≤74 years and HbA1c ≤8% for patients aged ≥75 years.
