# Supplementary material for: Adherence to diabetes quality indicators in primary care and all-cause mortality: A nationwide population-based historical cohort study
Source: PLoS One. 2024 May 9;19(5):e0302422. doi: 10.1371/journal.pone.0302422 (PMC11081362; doi:10.1371/journal.pone.0302422)
Supplement: S2 Table — (DOCX) [file pone.0302422.s005.docx]

**Table S2.** Baseline characteristics of the study population by adherence to process indicators in 2006. *

| **Influenza vaccination** | | **Eye clinic visit** | | **Urinary protein** | | **Serum creatinine** | | **Blood Pressure** | | **LDL-cholesterol** | | **HbA1c** | | **Variable** |
| --- | --- | --- | --- | --- | --- | --- | --- | --- | --- | --- | --- | --- | --- | --- |
| No | Yes | No | Yes | No | Yes | No | Yes | No | Yes | No | Yes | No | Yes |  |
| 138,082  (64.1) | 77,436  (35.9) | 84,592  (39.3) | 130,926  (60.7) | 67,805  (31.5) | 147,713  (68.5) | 20,060  (9.3) | 195,458  (90.7) | 46,843  (21.7) | 168,675  (78.3) | 24,419  (11.3) | 191,099  (88.7) | 23,216  (10.8) | 192,302  (89.2) | N  (%) |
| 52.9 | 49.8 | 49.9 | 53.0 | 52.2 | 51.6 | 46.1 | 52.4 | 48.2 | 52.8 | 46.3 | 52.5 | 47.4 | 52.3 | Female sex (%) |
| 64.1± 9.2 | 68.2±8.7 | 64.7±  9.6 | 66.1±  9.0 | 65.7± 9.7 | 65.5± 9.0 | 62.9± 9.3 | 65.8± 9.2 | 64.3± 9.5 | 65.9± 9.1 | 63.7± 9.7 | 65.8± 9.2 | 64.6± 9.8 | 65.7± 9.2 | Age (years) |
| 12.3 | 8.0 | 11.4 | 10.4 | 10.5 | 10.9 | 14.0 | 10.4 | 11.5 | 10.6 | 13.8 | 10.4 | 10.9 | 10.7 | Arabs (%) |
|  |  |  |  |  |  |  |  |  |  |  |  |  |  | SEP |
| 4.4 | 3.5 | 4.8 | 3.6 | 4.4 | 3.9 | 4.8 | 4.0 | 3.9 | 4.1 | 5.4 | 3.9 | 4.2 | 4.1 | 1-2 (low) |
| 51.3 | 43.1 | 50.1 | 47.2 | 48.8 | 48.1 | 51.1 | 48.1 | 46.1 | 49.0 | 51.2 | 48.0 | 49.6 | 48.2 | 3-5 |
| 39.0 | 45.4 | 39.3 | 42.6 | 40.6 | 41.6 | 38.4 | 41.6 | 42.8 | 40.9 | 37.9 | 41.7 | 39.8 | 41.5 | 6-8 |
| 5.3 | 8.1 | 5.8 | 6.6 | 6.1 | 6.3 | 5.8 | 6.3 | 7.2 | 6.0 | 5.5 | 6.4 | 6.5 | 6.2 | 9-10 (high) |
| 30.4 | 29.0 | 31.8 | 28.7 | 30.2 | 29.8 | 33.9 | 29.5 | 28.5 | 30.3 | 34.5 | 29.3 | 32.6 | 29.6 | Ever smokers (%) |
| 30.4± 6.3 | 29.9± 6.0 | 30.3± 6.5 | 30.2± 6.0 | 30.0± 6.3 | 30.4± 6.2 | 30.0± 6.2 | 30.3± 6.2 | 30.1± 6.5 | 30.3± 6.2 | 30.0± 6.0 | 30.3± 6.2 | 29.8± 6.1 | 30.3± 6.2 | BMI (kg/m^2^) |
| 37.9 | 39.1 | 38.4 | 38.3 | 38.9 | 38.1 | 40.2 | 38.1 | 39.1 | 38.1 | 39.3 | 38.2 | 39.8 | 38.2 | Overweight (%) |
| 29.2 | 27.9 | 28.3 | 28.9 | 27.9 | 29.1 | 28.1 | 28.8 | 28.7 | 28.7 | 28.4 | 28.7 | 27.6 | 28.8 | Obese (%) |

* Analyses included patients who survived 2006 (N=215,518). Values are expressed as percent except for plus–minus values are means ±SD. SEP: socioeconomic position, BMI: body mass index, Overweight: BMI 25.0-29.9, Obese: BMI ≥30.0 kg/m^2^, HbA1c: glycated hemoglobin, LDL-cholesterol: low density lipoprotein cholesterol.
