# Supplementary material for: Adherence to diabetes quality indicators in primary care and all-cause mortality: A nationwide population-based historical cohort study
Source: PLoS One. 2024 May 9;19(5):e0302422. doi: 10.1371/journal.pone.0302422 (PMC11081362; doi:10.1371/journal.pone.0302422)
Supplement: S3 Table — (DOCX) [file pone.0302422.s006.docx]

**Table S3.** Adjusted hazard ratio (95% CI) for mortality while excluding patients from the health maintenance organization in which documentations of blood pressure and influenza vaccination were unavailable for the baseline period, (N=174,327).

| #of years achieved indicator | **Process indicator** | | **Intermediate-outcome indicator** |
| --- | --- | --- | --- |
|  | Blood pressure | Influenza vaccination | Blood pressure  (≤140/90 mmHg) |
| 0 | 1.83 (1.69-1.98) | 1.03 (1.00-1.06) | 1.56 (1.49-1.63) |
| 1 | 1.86 (1.73-2.00) | 1.15 (1.12-1.19) | 1.36 (1.32-1.41) |
| 2 | 1.60 (1.51-1.69) | 1.18 (1.14-1.22) | 1.27 (1.23-1.31) |
| 3 | 1.21 (1.16-1.25) | 1.22 (1.18-1.26) | 1.13 (1.10-1.17) |
| 4 | 1.07 (1.04-1.09) | 1.18 (1.15-1.21) | 1.07 (1.04-1.10) |
| 5 | Reference | Reference | Reference |

Adjusted for age, gender, body mass index, socioeconomic position, smoking and health maintenance organization.
