# Supplementary material for: Adherence to diabetes quality indicators in primary care and all-cause mortality: A nationwide population-based historical cohort study
Source: PLoS One. 2024 May 9;19(5):e0302422. doi: 10.1371/journal.pone.0302422 (PMC11081362; doi:10.1371/journal.pone.0302422)
Supplement: S4 Table — (DOCX) [file pone.0302422.s007.docx]

**Table S4.** Baseline characteristics of the study population by adherence to intermediate-outcome indicators in 2006.

| **Blood Pressure**  **≤140/90 mmHg** | | **LDL-cholesterol**  **≤100 mg/dL** | | **HbA1c**  **(≤7%/ ≤8%) ^#^** | | **Variable** |
| --- | --- | --- | --- | --- | --- | --- |
| No | Yes | No | Yes | No | Yes |  |
| 49584  (29.4) | 119091  (70.6) | 83242  (43.6) | 107857  (56.4) | 91157  (47.4) | 101145  (52.6) | N  (%) |
| 55.3 | 51.7 | 56.7 | 49.3 | 51.8 | 52.7 | Female sex (%) |
| 66.9±8.9 | 65.5±9.2 | 65.3±9.3 | 66.3±9.0 | 62.9±8.2 | 68.2±9.2 | Age (years) |
| 10.4 | 10.6 | 12.3 | 8.9 | 15.7 | 6.3 | Arabs (%) |
|  |  |  |  |  |  | Socioeconomic position |
| 4.8 | 3.9 | 4.5 | 3.5 | 5.6 | 2.7 | 1-2 (low) |
| 50.2 | 48.5 | 51.4 | 45.3 | 52.1 | 44.7 | 3-5 |
| 39.2 | 41.6 | 38.8 | 44.0 | 37.4 | 45.2 | 6-8 |
| 5.8 | 6.1 | 5.3 | 7.2 | 4.9 | 7.4 | 9-10 (high) |
| 27.8 | 31.3 | 28.6 | 29.8 | 32.3 | 27.1 | Ever smokers (%) |
| 31.0±6.4 | 30.0±6.0 | 30.2±6.3 | 30.3±6.3 | 30.8±6.3 | 29.8±6.2 | BMI (kg/m2) |
| 35.9 | 39.1 | 38.1 | 38.4 | 36.3 | 39.9 | Overweight (%) |
| 30.2 | 28.1 | 28.4 | 28.9 | 30.3 | 27.5 | Obese (%) |

Analyses included patients who performed the test in 2006: N (HbA1c) =192,302, N (LDL-cholesterol) =191,099, N (Blood pressure) =168,675. Values are expressed as percent except for plus–minus values are means ±SD. BMI: body mass index, Overweight: BMI 25.0-29.9, Obese: BMI ≥30.0 kg/m^2^, HbA1c: glycated hemoglobin, LDL-cholesterol: low density lipoprotein cholesterol. ^#^ HbA1c ≤7% for patients aged ≤74 years and HbA1c ≤8% for patients aged ≥75 years.
