# Supplementary material for: Adherence to diabetes quality indicators in primary care and all-cause mortality: A nationwide population-based historical cohort study
Source: PLoS One. 2024 May 9;19(5):e0302422. doi: 10.1371/journal.pone.0302422 (PMC11081362; doi:10.1371/journal.pone.0302422)
Supplement: S5 Table — All the intermediate-outcome indicators in the same model, (N = 187,000). (DOCX) [file pone.0302422.s008.docx]

**Table S5.** Adjusted hazards ratio (95% CI) for mortality (2011-2016) by number of years with achieved target level (2006-2010). All the intermediate-outcome indicators in the same model, (N=187,000).

| LDL-cholesterol  (≤ 100 mg/dL) | Blood pressure  (≤ 140/90 mmHg) | HbA1c  (≤7%/ ≤8%) | Controlled years |
| --- | --- | --- | --- |
| 1.27 (1.23-1.31) | 1.33 (1.28-1.39) | 1.51(1.46-1.56) | 0 |
| 1.28 (1.24-1.32) | 1.26 (1.21-1.30) | 1.44 (1.39-1.49) | 1 |
| 1.20 (1.16-1.24) | 1.19 (1.15-1.23) | 1.38 (1.34-1.43) | 2 |
| 1.15 (1.12-1.18) | 1.08 (1.05-1.11) | 1.32 (1.27-1.36) | 3 |
| 1.07 (1.04-1.10) | 1.05 (1.02-1.07) | 1.22 (1.19-1.26) | 4 |
| REF | REF | REF | 5 |

Adjusted for age, gender, body mass index, socioeconomic position, smoking and health maintenance organization. Lack of measurement was considered as uncontrolled. HbA1c: glycated hemoglobin, HbA1c: HbA1c ≤7% among patients aged ≤74 years or HbA1c ≤8% among patients aged ≥75 years, LDL-cholesterol: low density lipoprotein cholesterol, CI: confidence interval.
