# Supplementary material for: Adherence to diabetes quality indicators in primary care and all-cause mortality: A nationwide population-based historical cohort study
Source: PLoS One. 2024 May 9;19(5):e0302422. doi: 10.1371/journal.pone.0302422 (PMC11081362; doi:10.1371/journal.pone.0302422)
Supplement: S6 Table — (DOCX) [file pone.0302422.s009.docx]

**Table S6.** Adjusted hazards ratio (95% CI) for mortality by number of years with achieved target level (2006-2010), stratified by gender N_Total_=187,000, N_Female_ =97,134, N_Male_ =89,866.

| LDL-cholesterol | | | Blood pressure | | | HbA1c (≤7%/≤8%) | | | HbA1c (≤9%) | | | Controlled years |
| --- | --- | --- | --- | --- | --- | --- | --- | --- | --- | --- | --- | --- |
| Female | Male | Total | Female | Male | Total | Female | Male | Total | Female | Male | Total |  |
| 1.52 (1.45-1.58) | 1.37 (1.30-1.44) | 1.45 (1.41-1.50) | 1.68 (1.59-1.78) | 1.35 (1.27-1.44) | 1.54 (1.47-1.60) | 1.75 (1.67-1.82) | 1.57 (1.51-1.64) | 1.66 (1.61-1.71) | 2.14 (2.01-2.27) | 1.88 (1.76-2.00) | 2.01 (1.92-2.10) | 0 |
| 1.46 (1.40-1.53) | 1.35 (1.28-1.41) | 1.41 (1.37-1.46) | 1.44 (1.37-1.51) | 1.31 (1.25-1.38) | 1.38 (1.34-1.43) | 1.56 (1.49-1.64) | 1.52 (1.45-1.59) | 1.55 (1.50-1.60) | 2.02 (1.91-2.14) | 1.88 (1.78-2.0) | 1.96 (1.88-2.04) | 1 |
| 1.36 (1.30-1.42) | 1.23 (1.18-1.29) | 1.30 (1.26-1.34) | 1.35 (1.29-1.41) | 1.18 (1.13-1.24) | 1.27 (1.23-1.31) | 1.55 (1.48-1.63) | 1.38 (1.31-1.45) | 1.47 (1.42-1.52) | 1.94 (1.84-2.03) | 1.72 (1.64-1.81) | 1.84 (1.77-1.90) | 2 |
| 1.25 (1.19-1.30) | 1.19 (1.14-1.24) | 1.22 (1.19-1.26) | 1.17 (1.13-1.22) | 1.10 (1.06-1.14) | 1.14 (1.11-1.17) | 1.43 (1.37-1.49) | 1.33 (1.27-1.39) | 1.38 (1.34-1.43) | 1.62 (1.55-1.69) | 1.55 (1.48-1.61) | 1.59 (1.55-1.64) | 3 |
| 1.12 (1.08-1.17) | 1.10 (1.05-1.14) | 1.11 (1.08-1.14) | 1.08 (1.04-1.12) | 1.00 (1.02-1.10) | 1.07 (1.04-1.10) | 1.29 (1.24-1.34) | 1.23 (1.17-1.28) | 1.26 (1.23-1.30) | 1.42 (1.38-1.47) | 1.31 (1.27-1.35) | 1.37 (1.34-1.40) | 4 |
| REF | REF | REF | REF | REF | REF | REF | REF | REF | REF | REF | REF | 5 |

Adjusted for age, body mass index, socioeconomic position, smoking and health maintenance organization. HbA1c: glycated hemoglobin, HbA1c: HbA1c ≤7% among patients aged ≤74 years or HbA1c ≤8% among patients aged ≥75 years, LDL-cholesterol: low density lipoprotein cholesterol, CI: confidence interval.
