# Supplementary material for: Adherence to diabetes quality indicators in primary care and all-cause mortality: A nationwide population-based historical cohort study
Source: PLoS One. 2024 May 9;19(5):e0302422. doi: 10.1371/journal.pone.0302422 (PMC11081362; doi:10.1371/journal.pone.0302422)
Supplement: S7 Table — (DOCX) [file pone.0302422.s010.docx]

**Table S7.** Adjusted hazards ratio (95% CI) for mortality by number of years with achieved target level (2006-2010), stratified by age group, N_Total_=187,000, N_age<65_ =95,619, N _age≥65_  = 91,381.

| LDL-cholesterol | | | Blood pressure | | | HbA1c (≤7%/≤8%) | | | HbA1c (≤9%) | | | Controlled years |
| --- | --- | --- | --- | --- | --- | --- | --- | --- | --- | --- | --- | --- |
| ≥65 | <65 | Total | ≥65 | <65 | Total | ≥65 | <65 | Total | ≥65 | <65 | Total |  |
| 1.42 (1.37-1.48) | 1.44 (1.35-1.54) | 1.45 (1.41-1.50) | 1.45 (1.38-1.52) | 1.76 (1.62-1.92) | 1.54 (1.47-1.60) | 1.69 (1.63-1.75) | 1.64 (1.53-1.77) | 1.66 (1.61-1.71) | 1.77 (1.66-1.88) | 2.30 (2.15-2.45) | 2.01 (1.92-2.10) | 0 |
| 1.39 (1.34-1.44) | 1.39 (1.30-1.48) | 1.41 (1.37-1.46) | 1.29 (1.24-1.35) | 1.67 (1.56-1.79) | 1.38 (1.34-1.43) | 1.59 (1.53-1.65) | 1.53 (1.41-1.66) | 1.55 (1.50-1.60) | 1.85 (1.76-1.95) | 2.14 (2.00-2.29) | 1.96 (1.88-2.04) | 1 |
| 1.31 (1.26-1.35) | 1.24 (1.16-1.32) | 1.30 (1.26-1.34) | 1.22 (1.18-1.26) | 1.42 (1.34-1.51) | 1.27 (1.23-1.31) | 1.54 (1.48-1.59) | 1.37 (1.25-1.49) | 1.47 (1.42-1.52) | 1.77 (1.69-1.85) | 1.98 (1.86-2.10) | 1.84 (1.77-1.90) | 2 |
| 1.24 (1.20-1.28) | 1.13 (1.06-1.20) | 1.22 (1.19-1.26) | 1.08 (1.04-1.11) | 1.32 (1.25-1.40) | 1.14 (1.11-1.17) | 1.43 (1.38-1.48) | 1.29 (1.17-1.41) | 1.38 (1.34-1.43) | 1.58 (1.53-1.63) | 1.63 (1.53-1.72) | 1.59 (1.55-1.64) | 3 |
| 1.12 (1.08-1.15) | 1.06 (0.99-1.13) | 1.11 (1.08-1.14) | 1.05 (1.02-1.09) | 1.13 (1.07-1.08) | 1.07 (1.04-1.10) | 1.28 (1.24-1.32) | 1.24 (1.13-1.36) | 1.26 (1.23-1.30) | 1.36 (1.33-1.40) | 1.40 (1.33-1.48) | 1.37 (1.34-1.40) | 4 |
| REF | REF | REF | REF | REF | REF | REF | REF | REF | REF | REF | REF | 5 |

Adjusted for age, gender, body mass index, socioeconomic position, smoking and health maintenance organization. HbA1c: glycated hemoglobin, HbA1c: HbA1c ≤7% among patients aged ≤74 years or HbA1c ≤8% among patients aged ≥75 years, LDL-cholesterol: low density lipoprotein cholesterol, CI: confidence interval.
