# Supplementary material for: Adherence to diabetes quality indicators in primary care and all-cause mortality: A nationwide population-based historical cohort study
Source: PLoS One. 2024 May 9;19(5):e0302422. doi: 10.1371/journal.pone.0302422 (PMC11081362; doi:10.1371/journal.pone.0302422)
Supplement: S8 Table — (DOCX) [file pone.0302422.s011.docx]

**Table S8.** Adjusted hazards ratio (95% CI) for mortality by number of years with achieved target level (2006-2010),
stratified by presence of cardiac disease.

| LDL-cholesterol | | | Blood pressure | | | HbA1c (≤7%/≤8%) | | | HbA1c (≤9%) | | | Controlled years |
| --- | --- | --- | --- | --- | --- | --- | --- | --- | --- | --- | --- | --- |
| 2011-2016 | Before 2011 | No | 2011-2016 | Before 2011 | No | 2011-2016 | Before 2011 | No | 2011-2016 | Before 2011 | No |  |
| 1.53 (1.40-1.68) | 1.56 (1.49-1.63) | 1.74 (1.64-1.85) | 1.63 (1.45-1.83) | 1.49 (1.40-1.57) | 1.79 (1.66-1.92) | 1.60 (1.46-1.74) | 1.61 (1.55-1.68) | 1.61 (1.52-1.70) | 2.0 (1.79-2.23) | 2.09 (1.96-2.22) | 2.03 (1.87-2.19) | 0 |
| 1.44 (1.31-1.58) | 1.51 (1.45-1.58) | 1.59 (1.50-1.69) | 1.33 (1.20-1.48) | 1.34 (1.28-1.41) | 1.54 (1.45-1.64) | 1.39 (1.26-1.53) | 1.54 (1.47-1.60) | 1.50 (1.41-1.60) | 1.81 (1.62-2.02) | 1.93 (1.83-2.04) | 2.11 (1.96-2.27) | 1 |
| 1.30 (1.18-1.43) | 1.36 (1.31-1.42) | 1.46 (1.37-1.54) | 1.32 (1.20-1.45) | 1.23 (1.18-1.28) | 1.36 (1.28-1.44) | 1.37 (1.2-1.51) | 1.46 (1.40-1.53) | 1.44 (1.35-1.53) | 1.68 (1.52-1.86) | 1.81 (1.73-1.90) | 1.90 (1.78-2.03) | 2 |
| 1.21 (1.10-1.32) | 1.28 (1.24-1.33) | 1.30 (1.23-1.38) | 1.22 (1.12-1.33) | 1.09 (1.05-1.13) | 1.21 (1.15-1.28) | 1.39 (1.27-1.53) | 1.39 (1.33-1.44) | 1.33 (1.25-1.41) | 1.47 (1.35-1.61) | 1.60 (1.54-1.66) | 1.62 (1.53-1.71) | 3 |
| 1.13 (1.03-1.24) | 1.14 (1.11-1.18) | 1.14 (1.08-1.21) | 1.17 (1.07-1.27) | 1.04 (1.00-1.07) | 1.12 (1.07-1.18) | 1.32 (1.21-1.44) | 1.27 (1.22-1.32) | 1.23 (1.16-1.29) | 1.39 (1.30-1.49) | 1.37 (1.33-1.41) | 1.38 (1.32-1.44) | 4 |
| REF | REF | REF | REF | REF | REF | REF | REF | REF | REF | REF | REF | 5 |

No: without cardiac disease, N=84,126. Before 2011: with cardiac disease before 2011, N=78,215. 2011-2016: complicated with cardiac disease during 2011-2016, N=24,659. HbA1c: glycated hemoglobin, HbA1c: HbA1c ≤7% among patients aged ≤74 years or HbA1c ≤8% among patients aged ≥75 years, LDL-cholesterol: low density lipoprotein cholesterol, CI: confidence interval. Adjusted for age, gender, body mass index, socioeconomic position, smoking and health maintenance organization.
