# Supplementary material for: Adherence to diabetes quality indicators in primary care and all-cause mortality: A nationwide population-based historical cohort study
Source: PLoS One. 2024 May 9;19(5):e0302422. doi: 10.1371/journal.pone.0302422 (PMC11081362; doi:10.1371/journal.pone.0302422)
Supplement: S9 Table — (DOCX) [file pone.0302422.s012.docx]

**Table S9.** Adjusted hazards ratio (95% CI) for mortality by number of years with achieved target level (2006-2010),
stratified by socioeconomic position.

| LDL-cholesterol | | | Blood pressure | | | HbA1c (≤7%/≤8%) | | | HbA1c (≤9%) | | | Controlled years |
| --- | --- | --- | --- | --- | --- | --- | --- | --- | --- | --- | --- | --- |
| SEP≥6 | SEP≤5 | Total | SEP≥6 | SEP≤5 | Total | SEP≥6 | SEP≤5 | Total | SEP≥6 | SEP≤5 | Total |  |
| 1.46 (1.39-1.53) | 1.38 (1.32-1.44) | 1.45 (1.41-1.50) | 1.62 (1.53-1.72) | 1.44 (1.36-1.53) | 1.54 (1.47-1.60) | 1.71 (1.63-1.79) | 1.61 (1.55-1.68) | 1.66 (1.61-1.71) | 1.86 (1.73-2.01) | 2.06 (1.95-2.17) | 2.01 (1.92-2.10) | 0 |
| 1.44 (1.37-1.51) | 1.34 (1.28-1.40) | 1.41 (1.37-1.46) | 1.43 (1.36-1.51) | 1.33 (1.27-1.39) | 1.38 (1.34-1.43) | 1.58 (1.50-1.66) | 1.53 (1.46-1.60) | 1.55 (1.50-1.60) | 2.07 (1.94-2.21) | 1.90 (1.80-2.00) | 1.96 (1.88-2.04) | 1 |
| 1.27 (1.21-1.33) | 1.28 (1.23-1.34) | 1.30 (1.26-1.34) | 1.27 (1.21-1.33) | 1.26 (1.21-1.32) | 1.27 (1.23-1.31) | 1.52 (1.45-1.60) | 1.43 (1.37-1.50) | 1.47 (1.42-1.52) | 1.86 (1.76-1.97) | 1.82 (1.74-1.91) | 1.84 (1.77-1.90) | 2 |
| 1.22 (1.17-1.27) | 1.19 (1.14-1.24) | 1.22 (1.19-1.26) | 1.15 (1.10-1.19) | 1.13 (1.08-1.17) | 1.14 (1.11-1.17) | 1.41 (1.35-1.48) | 1.37 (1.31-1.43) | 1.38 (1.34-1.43) | 1.65 (1.58-1.72) | 1.56 (1.50-1.62) | 1.59 (1.55-1.64) | 3 |
| 1.11 (1.06-1.15) | 1.09 (1.05-1.13) | 1.11 (1.08-1.14) | 1.10 (1.05-1.14) | 1.05 (1.02-1.09) | 1.07 (1.04-1.10) | 1.26 (1.21-1.31) | 1.27 (1.22-1.32) | 1.26 (1.23-1.30) | 1.37 (1.32-1.41) | 1.38 (1.34-1.42) | 1.37 (1.34-1.40) | 4 |
| REF | REF | REF | REF | REF | REF | REF | REF | REF | REF | REF | REF | 5 |

SEP: Socioeconomic position was defined based on the residential address, using scores ranging from 1 (lowest) to 10

(highest), N_Total_=187,000, N_SEP≤5_ = 9,6617, N _SEP≥6_ =9,6617. HbA1c: glycated hemoglobin, HbA1c: HbA1c ≤7% among patients aged ≤74 years or HbA1c ≤8% among patients aged ≥75 years, LDL-cholesterol: low density lipoprotein cholesterol, CI: confidence interval. Models were adjusted for age, gender, body mass index, socioeconomic position, smoking and health maintenance organization.
