# Supplementary material for: Adherence to diabetes quality indicators in primary care and all-cause mortality: A nationwide population-based historical cohort study
Source: PLoS One. 2024 May 9;19(5):e0302422. doi: 10.1371/journal.pone.0302422 (PMC11081362; doi:10.1371/journal.pone.0302422)
Supplement: S10 Table — (DOCX) [file pone.0302422.s013.docx]

**Table S10**. Adjusted hazards ratio (95% CI) for mortality (2007-2016) by the combined indicators in 2006, (A) among patients who were in follow-up and survived 2006 and (B) among those who were in follow-up and survived 2010.

|  |  | A Patients who were in follow-up and survived 2006 (N=215,518) | B Patients who were in  follow-up and survived 2010  (N= 187,000) |
| --- | --- | --- | --- |
|  |  |  |  |
| **HbA1c** | | | |
| Un-measured | N  HR (95% CI) | 23,216  1.51 (1.47-1.55) | 18,757  1.40 (1.35-1.44) |
| >7%/8%^#^ | N  HR (95% CI) | 91,157  1.31 (1.29-1.33) | 80,926  1.32 (1.30-1.35) |
| ≤7%/≤8%^#^ | N  HR (95% CI) | 101,145  Reference | 87,317  Reference |
| **LDL-cholesterol** | |  |  |
| Un-measured | N  HR (95% CI) | 26,099  1.42 (1.39-1.45) | 21,544  1.33 (1.29-1.37) |
| >100 mg/dL | N  HR (95% CI) | 83,242  1.10 (1.08-1.12) | 72,157  1.08 (1.06-1.10) |
| ≤100 mg/dL | N  HR (95% CI) | 106,177  Reference | 93,299  Reference |
| **Blood pressure** | |  |  |
| Un-measured | N  HR (95% CI) | 46,843  1.19 (1.17-1.22) | 39,962  1.13 (1.10-1.16) |
| > 140/90 mmHg | N  HR (95% CI) | 49,584  1.08 (1.06-1.10) | 42,883  1.13 (1.11-1.16) |
| ≤ 140/90 mmHg | N  HR (95% CI) | 119,091  Reference | 104,155  Reference |

HbA1c: glycated hemoglobin, ^#^ HbA1c: 7% among patients aged ≤74 years and 8% among patients aged ≥75 years, LDL-cholesterol: low density lipoprotein cholesterol, CI: confidence interval. Models were adjusted for age, gender, body mass index, socioeconomic position, smoking and health maintenance organization.
